# Author Correction: Aberrant activity of mitochondrial NCLX is linked to impaired synaptic transmission and is associated with mental retardation

**DOI:** 10.1038/s42003-021-02312-w

**Published:** 2021-06-14

**Authors:** Alexandra Stavsky, Ohad Stoler, Marko Kostic, Tomer Katoshevsky, Essam A. Assali, Ivana Savic, Yael Amitai, Holger Prokisch, Steffen Leiz, Cornelia Daumer-Haas, Ilya Fleidervish, Fabiana Perocchi, Daniel Gitler, Israel Sekler

**Affiliations:** 1 Department of Physiology and Cell Biology, Faculty of Health Sciences, Ben-Gurion University of the Negev, Beer-Sheva, Israel; 2 Zlotowski Center for Neuroscience, Ben-Gurion University of the Negev, Beer-Sheva, Israel; 3 Institute of Human Genetics, School of Medicine, Technische Universität München, Munich, Germany; 4 Institute of Neurogenomics, Helmholtz Zentrum München, Munich, Germany; 5 Department of Pediatrics, Klinikum Dritter Orden, Munich, Germany; 6 Pränatal-Medizin München, Munich, Germany; 7 Institute for Diabetes and Obesity, Helmholtz Diabetes Center (HDC), Helmholtz Zentrum München, Munich, Germany; 8 Munich Cluster for Systems Neurology, Munich, Germany

**Keywords:** Synaptic transmission, Mitochondria, Calcium signalling, Cellular neuroscience

Correction to: *Communications Biology* 10.1038/s42003-021-02114-0, published online 2 June 2021

The original version of this Article contained an error in the spelling of the author Fabiana Perocchi, which was incorrectly given as Fabiana Perrochi. This has now been corrected in both the PDF and HTML versions of the Article.

