# Supplementary material for: Aberrant activity of mitochondrial NCLX is linked to impaired synaptic transmission and is associated with mental retardation
Source: Commun Biol. 2021 Jun 2;4:666. doi: 10.1038/s42003-021-02114-0 (PMC8172942; doi:10.1038/s42003-021-02114-0)
Supplement: Supplementary file 1 — Supplementary Information [file 42003_2021_2114_MOESM1_ESM.pdf]

Supplementary Figures and Legends

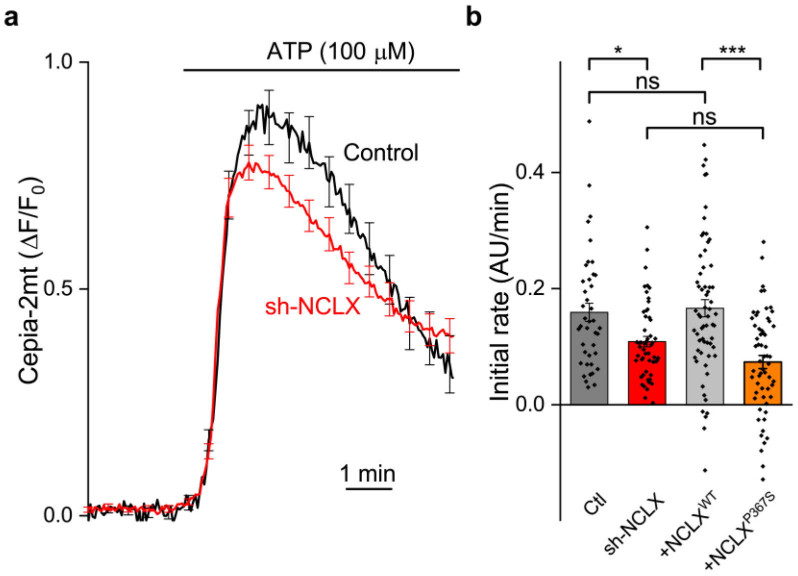

Supplementary Fig. 1: Knock-down of NCLX in SH-SY5Y cells slows mitochondrial calcium efflux.

a *Cepia2-mt was expressed in SH-SY5Y cells (black) or in cells in which the endogenous NCLX was knocked down by shRNA (red), and mitochondrial transients (Cepia2-mt fluorescence) were induced by bath application of ATP (horizontal line). Shown are mean±SEM traces (n=41 and 53, respectively).*

b *Linear fit of a 150 seconds period after calcium levels started to decline served to determine the initial calcium efflux rate. Shown are extrusion rates extracted from data in graph in **a** and Fig. 1**f** (0.159±0.016, 0.109±0.009, 0.166±0.016, 0.073±0.011 arbitrary units/minute). Efflux rates differed between the experimental groups (Kruskal Wallis ANOVA, *** p=3e-6, chi-square=28.72, DF=3; post-hoc pairwise comparisons using the Mann-Whitney u-test with Bonferroni's correction). KD of NCLX reduced the efflux rate compared to control (* p=0.048), which was rescued by reintroduction of WT NCLX (p>0.05 vs. control), but not by the P367S variant (p>0.05 vs. KD), for which the efflux rate was significantly lower than compared to WT NCLX (*** p<0.001).*

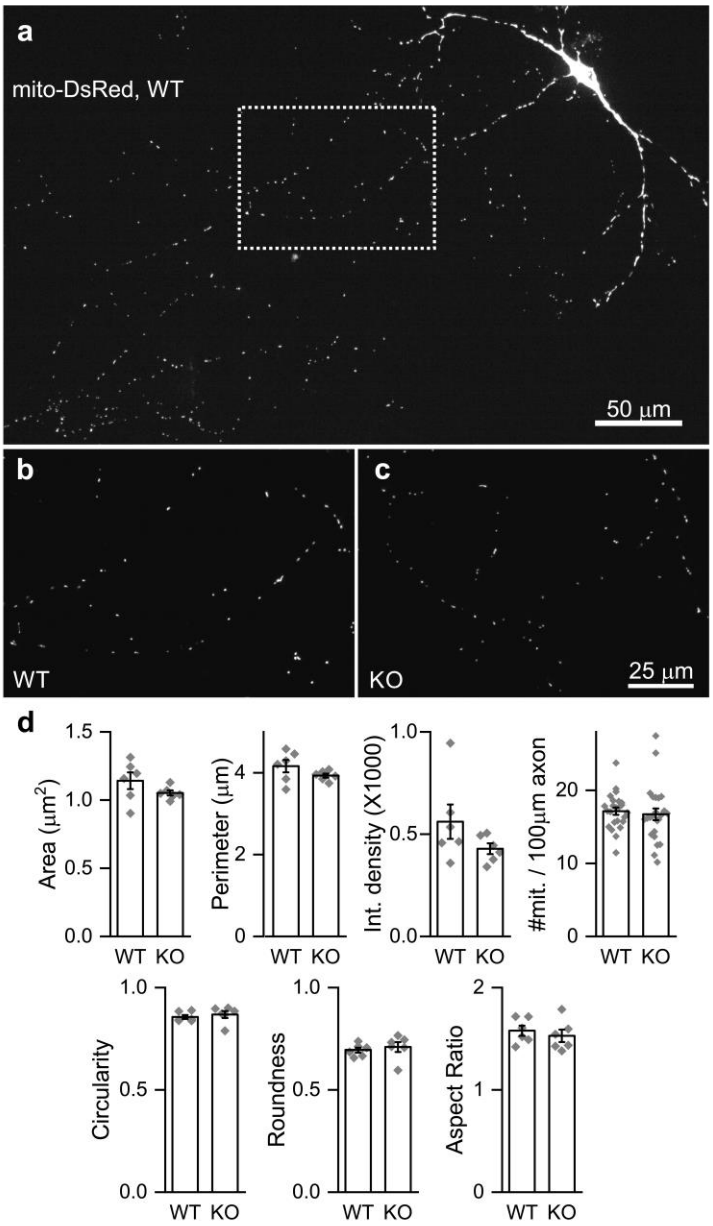

Supplementary Fig. 2: Deletion of NCLX does not alter mitochondrial morphology in axons of cultured hippocampal neurons.

a *Cultured hippocampal neurons were sparsely transfected with plasmids for the expression mito-DsRed, and were imaged at DIV 14 to visualize mitochondria. Representative image of a WT neuron, showing the cell body, primary dendrites and an axonal arbor.*

b *Magnification of rectangle in* ***a****. Shown are axonal mitochondria, identified by their compact size and by their localization in a neurite far from the cell body.*

c *Representative image of axonal mitochondria in an NCLX KO neuron, to the same scale as in* ***b****.*

d *Quantification of parameters related to mitochondrial size (area, perimeter length, integrated density), number of mitochondria per 100μm of axon, and shape (circularity, roundness, long to short axis aspect ratio). Symbols represent the average of each parameter in independent pictures (n=6,6 pictures of WT and KO axons, 80-500 mitochondria per picture), except for mitochondrial counts, for which n=24,24. Bars and error bars represent mean±SEM values. No significant differences were observed between the genotypes for all properties (p>0.16-0.65, two-tailed Students' t-tests).*

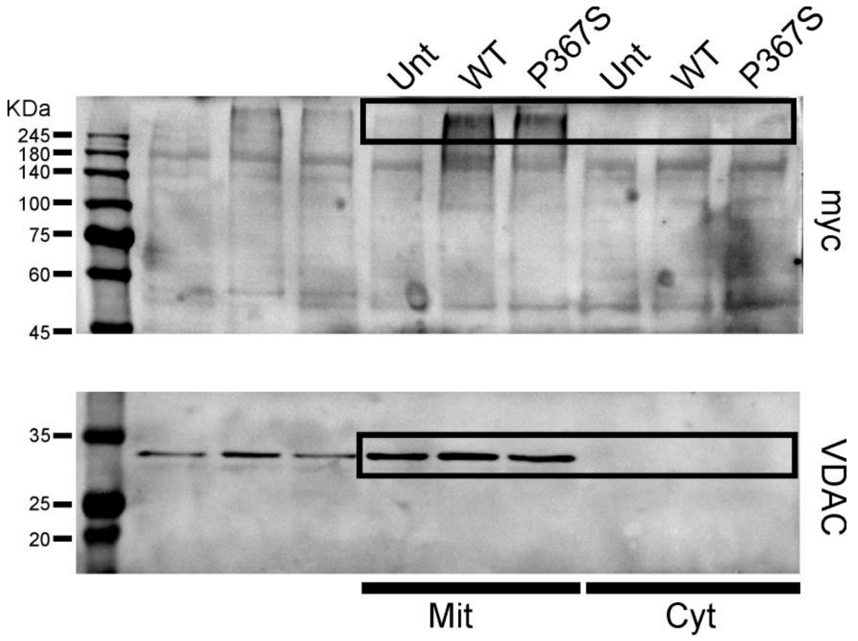

Supplementary Fig. 3: Full blots of data shown in Fig. 1d. *Unt: untransfected, WT: myc-NCLX^{WT}, P367S: myc-NCLX^{P367S}, Mit: mitochondrial fraction, Cyt: cytoplasmic fraction, KDa: Kilo Dalton. Primary antibody indicated on right. Boxes denote the blots shown in Fig. 1d. Panels originate from the same blot, which was cut just below the 45 KDa marker.*
