# Supplementary material for: Aberrant activity of mitochondrial NCLX is linked to impaired synaptic transmission and is associated with mental retardation
Source: Commun Biol. 2021 Jun 2;4:666. doi: 10.1038/s42003-021-02114-0 (PMC8172942; doi:10.1038/s42003-021-02114-0)
Supplement: Supplementary file 3 — Description of Additional Supplementary Files [file 42003_2021_2114_MOESM3_ESM.pdf]

Description of Additional Supplementary Files

File Name: Supplementary Data 1
Description: Source data for the graphs and charts is available as a Supplementary Data excel file
