# Supplementary material for: Aberrant activity of mitochondrial NCLX is linked to impaired synaptic transmission and is associated with mental retardation
Source: Commun Biol. 2021 Jun 2;4:666. doi: 10.1038/s42003-021-02114-0 (PMC8172942; doi:10.1038/s42003-021-02114-0)
Supplement: Supplementary file 5 — Reporting Summary [file 42003_2021_2114_MOESM5_ESM.pdf]

nature research

Corresponding author(s): Daniel Gitler and Israel Sekler

Last updated by author(s): Jan 27, 2021

Reporting Summary

Nature Research wishes to improve the reproducibility of the work that we publish. This form provides structure for consistency and transparency in reporting. For further information on Nature Research policies, see our Editorial Policies and the Editorial Policy Checklist.

Statistics

For all statistical analyses, confirm that the following items are present in the figure legend, table legend, main text, or Methods section.

n/a	Confirmed	
☐	☒	The exact sample size (*n*) for each experimental group/condition, given as a discrete number and unit of measurement
☐	☒	A statement on whether measurements were taken from distinct samples or whether the same sample was measured repeatedly
☐	☒	The statistical test(s) used AND whether they are one- or two-sided<br>*Only common tests should be described solely by name; describe more complex techniques in the Methods section.*
☐	☒	A description of all covariates tested
☐	☒	A description of any assumptions or corrections, such as tests of normality and adjustment for multiple comparisons
☐	☒	A full description of the statistical parameters including central tendency (e.g. means) or other basic estimates (e.g. regression coefficient) AND variation (e.g. standard deviation) or associated estimates of uncertainty (e.g. confidence intervals)
☐	☒	For null hypothesis testing, the test statistic (e.g. *F*, *t*, *r*) with confidence intervals, effect sizes, degrees of freedom and *P* value noted<br>*Give P values as exact values whenever suitable.*
☒	☐	For Bayesian analysis, information on the choice of priors and Markov chain Monte Carlo settings
☒	☐	For hierarchical and complex designs, identification of the appropriate level for tests and full reporting of outcomes
☒	☐	Estimates of effect sizes (e.g. Cohen's *d*, Pearson's *r*), indicating how they were calculated

Our web collection on statistics for biologists contains articles on many of the points above.

Software and code

Policy information about availability of computer code

Data collection	NIS elements 5.1, pClamp 10.0
Data analysis	Originlab 2018, 2020, NIS elements 5.1, imageJ (WEKA)

For manuscripts utilizing custom algorithms or software that are central to the research but not yet described in published literature, software must be made available to editors and reviewers. We strongly encourage code deposition in a community repository (e.g. GitHub). See the Nature Research guidelines for submitting code & software for further information.

Data

Policy information about availability of data

All manuscripts must include a data availability statement. This statement should provide the following information, where applicable:

- Accession codes, unique identifiers, or web links for publicly available datasets
- A list of figures that have associated raw data
- A description of any restrictions on data availability

The datasets generated during and/or analyzed during the current study are available from the corresponding authors on reasonable request.

Field-specific reporting

Life sciences study design

All studies must disclose on these points even when the disclosure is negative.

Sample size	Sample size was chosen such that each condition was sampled from at least 3 independent sources (mice, cell cultures), each producing >=3 products (slices, coverslips etc). Each cover slip was sampled once. Each sampling (image) contained multiple measurable units (synapses, cells), numbers as indicated.
Data exclusions	Grubbs's test was used to identify outliers in otherwise normally distributed data sets. Experiments that were deemed to have failed on technical grounds (no responses recorded or similar) were excluded in their entirety.
Replication	Experiments were performed independently at least 3 times (3 independent cultures, 3 independent infections etc) and the similarity of results across similar experimental conditions was tested to assess reproducibility.
Randomization	Experimental design was not random. Comparisons were performed between materials obtained from WT and knockout mice. Knockout mice were back-crossed onto the C57Bl6 background.
Blinding	Experiments did not follow a blind design. However, when applicable the choice of regions of interest for analysis was performed on images that do not include the measured quantity (for example, the red calcium-invariant channel in calcium imaging experiments or baseline images prior to treatments), or from images in series, in which the measured quantity is not evident (final values at completion of sypHy experiments)

Reporting for specific materials, systems and methods

We require information from authors about some types of materials, experimental systems and methods used in many studies. Here, indicate whether each material, system or method listed is relevant to your study. If you are not sure if a list item applies to your research, read the appropriate section before selecting a response.

Materials & experimental systems

n/a	Involved in the study	
☐	☒	Antibodies
☐	☒	Eukaryotic cell lines
☒	☐	Palaeontology and archaeology
☐	☒	Animals and other organisms
☐	☒	Human research participants
☒	☐	Clinical data
☒	☐	Dual use research of concern

Methods

n/a	Involved in the study	
☒	☐	ChIP-seq
☒	☐	Flow cytometry
☒	☐	MRI-based neuroimaging

Antibodies

Antibodies used	Goat polyclonal anti vGlut1, Synaptic Systems (135 307); Goat polyclonal anti-VDAC1, Santa Cruz Biotechnology (sc-8828); anti-myc, Abcam (ab18185)
Validation	vGlut1 antibody validated by manufacturer in knockout tissue;

Eukaryotic cell lines

Policy information about cell lines

Cell line source(s)	ATCC
Authentication	By supplier
Mycoplasma contamination	Periodic testing was performed for mycoplasma
Commonly misidentified lines (See ICLAC register)	N/A

Animals and other organisms

Policy information about studies involving animals; ARRIVE guidelines recommended for reporting animal research

Laboratory animals	Mice: WT - C57BL/6JRccHsd (Envigo), NCLX-KO; Slc8b1em1J (Jackson laboratories) of either sex. For cultures, pups of age P0-P2 were used. For slice recordings, mice of age P18-P21 were used
Wild animals	The study did not involve wild animals

Field-collected samples	The study did not involve samples collected from the field
Ethics oversight	Ben-Gurion University committee for the ethical care and use of animals in experiments

Note that full information on the approval of the study protocol must also be provided in the manuscript.

Human research participants

Policy information about studies involving human research participants

Population characteristics	One family
Recruitment	Research participant arrived as patients to the clinic for consultation
Ethics oversight	The ethical committee of the Technical University of Munich

Note that full information on the approval of the study protocol must also be provided in the manuscript.
